# Operationssaalmanagement in einem Haus der Maximalversorgung vor und während der COVID-19-Pandemie

**DOI:** 10.1007/s00104-025-02385-x

**Published:** 2025-10-07

**Authors:** Vladan Tenic, Jörg Heckenkamp, Jelena Raskovic, Andreas Hoene, Reinhart T. Grundmann

**Affiliations:** 1 Department for Vascular Medicine, Marienhospital Osnabrück, Osnabrück, Deutschland; 2 Faculty of Economics and Business, University of Belgrade, Belgrade, Serbien; 3 Clinic for General, Visceral, Vascular and Thoracic Surgery, University Medicine Greifswald, Greifswald, Deutschland; 4 University Heart and Vascular Center Hamburg, Department for Vascular Medicine, University Hospital Hamburg-Eppendorf, Hamburg, Deutschland

**Keywords:** COVID-19, Operationssaal-Nutzung, Operationszeit, Wechselzeit, Schwerpunktkrankenhaus, COVID 19, Operating room uzilization, Operating time, Turnover time, Tertiary care center

## Abstract

**Hintergrund:**

Die COVID-19-Pandemie führte zu einer Einschränkung des Operationsbetriebs.

**Fragestellung:**

Wie wirkte sich die COVID-19-Pandemie über 3 Jahre auf Fallaufkommen und Effizienz des Operationsbetriebs in einem Haus der Maximalversorgung aus?

**Material und Methodik:**

Im Zeitraum vom 01.01.2019 bis 31.07.2022 wurde die Nutzung von 6 Operationssälen jeweils in den ersten 7 Monaten (Januar bis Juli) während des Tagesbetriebs (8:00 bis 16:00 Uhr) von Montag bis Freitag erfasst. Bestimmt wurden Operationszahl, chirurgische Nutzungszeit, Wechselzeit, Startzeit und Operationsschluss. Zusätzlich wurde die Zahl der im Krankenhaus behandelten COVID-19-Patienten dokumentiert. Es handelte sich insgesamt um 10.193 konsekutive Eingriffe.

**Ergebnisse:**

Die Operationszahl sank 2020 um 14,9 %, 2021 um 23,1 % und 2022 um 16,7 % im Vergleich zu 2019. Die chirurgische Nutzungszeit lag 2020 13,7 %, 2021 20,8 % und im Jahr 2022 13,5 % unter dem Wert von 2019. Die Wechselzeit pro Eingriff und die Operationszeit pro Eingriff nahmen signifikant zu. Die Startzeit pro Tag und Saal verzögerte sich signifikant, der Operationsschluss war signifikant früher. Mit steigender Zahl stationär behandelter COVID-19-Patienten nahm die Operationszahl signifikant ab (*p* = 0,002). Mit jedem zusätzlichen COVID-19-Patienten verringerte sich die chirurgische Nutzungszeit der Operationssäle im Durchschnitt um etwa 172,99 min (*p* < 0,001) und die verspätete Startzeit stieg im Mittel um 46,79 min pro Monat an (*p* = 0,000).

**Folgerung:**

Die COVID-19-Pandemie führte, verglichen mit 2019, bis in das Jahr 2022 zu einer signifikanten Reduktion des Operationsaufkommens, verringerter Operationssaalnutzung und erhöhten Wechselzeiten in einem Haus der Maximalversorgung bei gefäßchirurgischen, viszeralchirurgischen und unfallchirurgischen Eingriffen.

**Graphic abstract:**

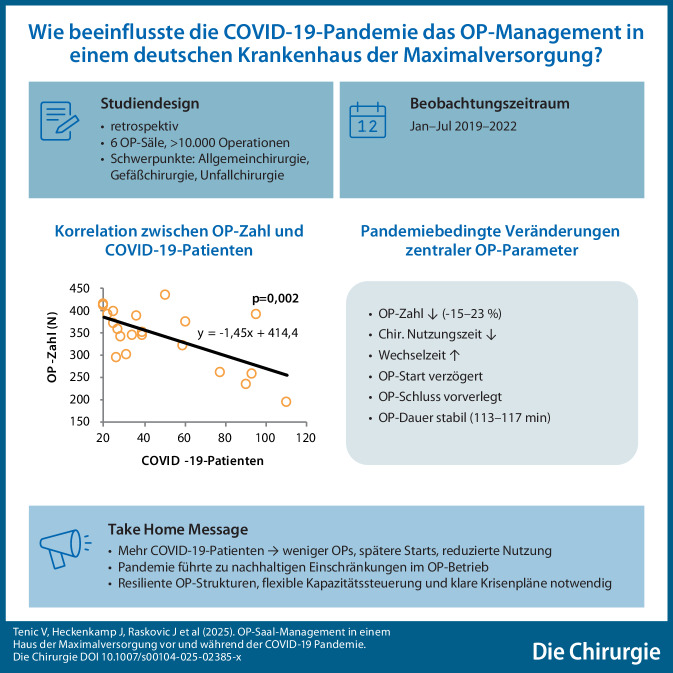

## Abstract (en)

**Background:**

The coronavirus disease 2019 (COVID-19) pandemic led to significant restrictions in surgical services.

**Objective:**

To investigate the impact of the COVID-19 pandemic over 3 years on surgical case volume and operating room (OR) efficiency in a tertiary care hospital.

**Material and methods:**

From 1 January 2019 to 31 July 2022, the use of 6 ORs was analyzed for the first 7 months of each year (January–July), limited to regular daytime hours (8:00–16:00) from Monday to Friday. Key metrics included the number of surgeries, surgical utilization time, turnover time, start time and end time. Additionally, the number of inpatients with confirmed COVID-19 was documented. In total, 10,193 consecutive procedures were evaluated.

**Results:**

Compared to 2019, the number of surgeries decreased by 14.9% in 2020, 23.1% in 2021, and 16.7% in 2022. Surgical utilization time declined by 13.7% in 2020, 20.8% in 2021, and 13.5% in 2022. Turnover time and operating time per procedure significantly increased. The OR start times were significantly delayed, while end times occurred earlier. A significant inverse correlation was found between the number of hospitalized COVID-19 patients and surgical volume (*p* = 0.002). For each additional COVID-19 inpatient, surgical utilization time decreased by an average of 172.99 min (*p* < 0.001) and the delay in daily start time increased on average by 46.79 min per month (*p* = 0.000).

**Conclusion:**

The COVID-19 pandemic led to a sustained reduction in surgical case volume, decreased OR utilization and increased turnover times through 2022 in a tertiary care center, affecting vascular, visceral and trauma surgery.

## Hintergrund

Über die Auswirkungen der COVID-19-Pandemie auf das Management chirurgischer Krankheitsbilder ist in Deutschland ausführlich berichtet worden, so bei kolorektaler Chirurgie [13], hepatopankreatobiliärer Chirurgie und Organtransplantation [10], onkologischer Chirurgie des oberen Gastrointestinaltrakts [2] oder bei der Versorgung bariatrischer Patienten [19]. Auch wurden Ressourcenbedarf bei chirurgischer Behandlung von COVID-19-Patienten [11] und ökonomische Auswirkungen der nichtselektiven Einschränkung des elektiven Operationsbetriebs während der COVID-19-Pandemie dargestellt [3]. Daten zum Operationssaalmanagement per se, vor, während und nach der COVID-19-Pandemie sind hingegen rar. Fischer et al. [9] berichteten lediglich, dass sich die Operationszeiten bei Cholezystektomien während der COVID-19-Pandemie von 64 min (SD 34 min) auf durchschnittlich 71 min (SD 38 min) signifikant verlängerten (*p* = 0,01). Ausführlicher war eine Analyse des Operationsbetriebs im Universitätsklinikum Ulm während der Pandemie [7]. Diese Arbeitsgruppe fand im Vergleich zum Referenzjahr 2019 im Jahr 2020 eine Abnahme des Fallvolumens um 31 % und im Jahr 2021 um 23 %. Gleichzeitig kam es zu erheblichen Zeitverzögerungen im Operationssaalmanagement. Die mittlere Aufenthaltsdauer des Patienten im Operationssaal („Säulenzeit“) beispielsweise stieg von 65 min (2019) auf 87 min (2020) an und war auch noch 2021 (72 min) und 2022 (74 min) deutlich erhöht. Die Autoren folgerten, dass die durch die COVID-19-Pandemie bedingten chirurgischen Einschränkungen die operative Leistung in ihrem Klinikum langfristig verringert haben.

Einer ähnlichen Fragestellung geht die vorliegende Untersuchung nach. Es sollte geklärt werden, wie sich die COVID-19-Pandemie auf Fallaufkommen und Effizienz des Operationsbetriebs in einem Haus der Maximalversorgung im Vergleich zu der Zeit vor der Pandemie (Jahr 2019) bis zur weitgehenden Beendigung der bundesweiten Eindämmungsmaßnahmen im ersten Halbjahr 2022 ausgewirkt hat. Dabei erfassten wir die wesentlichen Messparameter der Operationseffizienz, wie sie auch der National Health Service (NHS) empfiehlt [5]: Startzeit, Saalauslastung, Zahl der Operationen und Zeitlücke zwischen den Operationen. Inwieweit diese Parameter mit der Zahl der im Krankenhaus zum gleichen Zeitraum behandelten COVID-19-Patienten korrelierten, sollte hier erstmals überprüft werden.

## Methodik

Im Zeitraum vom 01.01.2019 bis 31.07.2022 wurde im Marienhospital Osnabrück (Krankenhaus der Maximalversorgung) die Nutzung von 6 Operationssälen jeweils in den ersten 7 Monaten (Januar bis Juli) während des Tagesbetriebs (8:00–16:00 Uhr) von Montag bis Freitag erfasst. Damit konnten vier identische Zeitperioden in einem Abstand von jeweils einem Jahr verglichen werden. Folgende Parameter wurden bestimmt:

1. Operationszahl: die Gesamtzahl der chirurgischen Eingriffe (monatlich/Jahresperiode).
2. Chirurgische Nutzungszeit: die Zeit von der Einschleusung des Patienten in den Operationssaal bis zu seiner Entlassung aus dem Operationssaal.
3. Wechselzeit: die Zeit zwischen zwei Eingriffen (Zeit zwischen Entlassung des ersten Patienten aus dem Operationssaal und Einschleusung des nächsten Patienten in den Operationssaal).
4. Operationszeit: die Gesamtzeit der chirurgischen Nutzungszeit (monatlich/Jahresperiode) einschließlich Eingriffe außerhalb des Regelbetriebes.
5. Startzeit: der Zeitpunkt des Beginns des ersten chirurgischen Eingriffs (Patient im Saal).
6. Operationsschluss: der Zeitpunkt des Abschlusses des letzten chirurgischen Eingriffs (Patient aus dem Saal).
7. Zahl der im Marienhospital behandelten COVID-19-Patienten auf den Allgemeinstationen und auf der Intensivstation (monatlich/Jahresperiode).

Die chirurgische Nutzungszeit bezieht sich auf den Regelbetrieb (8:00 bis 16:00 Uhr).

Die Operationszeit pro Tag erfasste über den Regelbetrieb hinaus alle Eingriffe, die vor 16:00 Uhr begonnen und nach 16:00 Uhr fortgesetzt wurden. Die Daten wurden den Operationsprotokollen entnommen und zur Auswertung in Excel-Tabellen übertragen.

### Operationssäle

Erfasst wurde die Nutzung von 6 Operationssälen, die folgenden Disziplinen zugeordnet waren:

- Saal 1 und Saal 2 – überwiegend Gefäßchirurgie
- Saal 3 und Saal 4 – überwiegend Viszeralchirurgie
- Saal 5 und Saal 6 – Unfallchirurgie gemischt mit Neurochirurgie.

### Statistik

Bevor mit der Datenanalyse begonnen wurde, wurde geprüft, ob die Datenverteilung für jedes Jahr signifikant von der Normalverteilung abwich. Eine Normalverteilung der Daten war eine Voraussetzung für die Anwendung parametrischer Auswertungsmethoden. Falls die Daten stark von der Normalverteilung abwichen, wurden nichtparametrische Methoden zur Auswertung angewendet. Zur Überprüfung der Normalverteilung wurde der Kolmogorov-Smirnov-Test benutzt.

Zu den angewandten parametrischen Methoden gehörte der Vergleich der Mittelwerte (Means) zwischen zwei Jahren. Es handelte sich um den Vergleich unabhängiger Gruppen. Hier wurden zwei Tests verwendet: einfacher t‑Test, falls die Varianzen zwischen den Gruppen (Jahren) homogen waren, und Welch-t-Test, falls die Varianzen nicht homogen waren. Falls der *p*-Wert < 0,05 lag, galt der Unterschied als signifikant.

Wenn die Daten nicht normalverteilt waren, kamen nichtparametrische Tests zur Anwendung: Mann-Whitney-U-Test – vergleicht Mediane anstatt Mittelwerte (Means) und der Kolmogorov-Smirnov-Test – untersucht die Differenz zwischen Verteilungen. Die Differenz basiert auf der größten absoluten Differenz zwischen den kumulativen Verteilungen der Gruppen.

Falls Extremwerte die Mediane beeinflussten und die nichtparametrischen Tests irreführende Ergebnisse lieferten, wurde das Linear Mixed Model (LMM) verwendet.

## Ergebnisse

### Operationssaalnutzung, Wechselzeiten und Operationszeiten

Eine Übersicht über die untersuchten Parameter zur Nutzung der Operationssäle in den Jahren 2019 bis 2022 gibt Tab. 1. Es lassen sich deutliche Auswirkungen der COVID-19-Pandemie auf die verschiedenen operativen Kennzahlen erkennen. Operationszahl, chirurgische Nutzungszeit und Operationszeit zeigen während der Pandemiejahre signifikante Rückgänge im Vergleich zum Referenzjahr 2019.

**Tab. 1:** Nutzung und Auslastung der Operationssäle (2019–2022) – Gesamtübersicht

Jahr	2019	2020	2021	2022
COVID-19-Patienten (*N*)	0	287	475	244
Operationszahl (*N*)	2952	2511	2270	2460
	(100 %)	(85,06 %)	(76,90 %)	(83,33 %)
*p‑Wert*	*Referenz*	*0,000*	*0,000*	*0,000*
Chirurgische Nutzungszeit (min)	310.505	267.969	246.042	268.609
	(100 %)	(86,30 %)	(79,24 %)	(86,51 %)
*p‑Wert*	*Referenz*	*0,000*	*0,000*	*0,000*
Wechselzeit (min)	68.357	59.218	59.597	71.218
	(100 %)	(86,63 %)	(87,18 %)	(104,19 %)
*p‑Wert*	*Referenz*	*0,003*	*0,000*	*0,000*
Operationszeit (min)	334.940	289.567	267.584	289.693
	(100 %)	(86,45 %)	(79,89 %)	(86,49 %)
*p‑Wert*	*Referenz*	*0,220*	*0,003*	*0,002*
Startzeit (min nach 8:00 Uhr)	18.382	29.949	44.260	35.631
	(100 %)	(162,93 %)	(240,78 %)	(193,84 %)
*p‑Wert*	*Referenz*	*0,000*	*0,000*	*0,000*
Operationsschluss (min vor/nach 16:00 Uhr)	1842	−6738	−3420	−13.979
	(100 %)	(465,80 %)	(285,67 %)	(858,90 %)
*p‑Wert*	*Referenz*	*0,159*	*0,728*	*0,003*

Die Operationszahl sank 2020 um 14,9 %, 2021 um 23,1 % und erholte sich 2022 nur leicht mit einem Rückgang von 16,7 % im Vergleich zu 2019. Der tiefste Stand wurde somit im Jahr 2021 erreicht. Die chirurgische Nutzungszeit verringerte sich ebenfalls deutlich: 2020 betrug der Rückgang 13,7 %, 2021 sogar 20,8 %. Im Jahr 2022 lag die Nutzungszeit immer noch 13,5 % unter dem Wert von 2019. Auch die Operationszeit zeigte einen kontinuierlichen Rückgang: 2020 sank sie um 13,6 %, 2021 um 20,1 % und 2022 um 13,5 %.

Ein differenziertes Bild ergab sich bei der Wechselzeit: 2020 und 2021 lag diese jeweils um 13,4 % bzw. 12,8 % unter dem Wert von 2019. Im Jahr 2022 hingegen stieg die Wechselzeit auf 104,2 % des Ausgangswerts an, was auf verlängerte Vorbereitungszeiten schließen lässt.

Die Startzeit (Operationsbeginn) verzögerte sich während der Pandemie deutlich. Im Vergleich zu 2019 (100 %) stieg sie im Jahr 2020 auf 162,9 %, 2021 auf 240,8 % und 2022 auf 193,8 % an. Das bedeutet eine Verdopplung der Verspätung gegenüber dem Ausgangsniveau.

Der Operationsschluss lag 2019 bei 1842 min (über 16:00 Uhr Regelbetrieb), er verkürzte sich 2020 auf −6738 min. Auch 2021 (−3420 min) und 2022 (−13.979 min) lagen die Werte deutlich unter dem Referenzwert, was eine vorzeitige Beendigung des Regelbetriebs belegt.

Einen Überblick über Nutzung und Auslastung der Operationssäle in den einzelnen Jahren, bezogen auf Operationsanzahl und chirurgische Nutzungszeit pro Saal, Wechselzeit pro Eingriff, Operationszeit pro Eingriff sowie Startzeit und Operationsschluss pro Tag und Saal gibt Tab. 2. Wie ersichtlich, nahm die Zahl der Eingriffe pro Saal und die chirurgische Nutzungszeit während der Pandemie signifikant ab. Die Wechselzeit pro Eingriff nahm signifikant zu, geringer auch die Operationszeit pro Eingriff. Die Startzeit pro Tag und Saal verzögerte sich signifikant, der Operationsschluss pro Tag und Saal war signifikant früher. Alle Werte erreichten auch noch im Jahr 2022 nicht wieder das Ausgangsniveau.

**Tab. 2:** Nutzung und Auslastung der Operationssäle (2019–2022) – Mittelwerte

Jahr	2019	2020	2021	2022
Operationszahl (*N*)	3,37	2,85	2,59	2,81
*Pro Tag pro Saal*	(100 %)	(84,57 %)	(76,85 %)	(83,38 %)
*p‑Wert*	*Referenz*	*0,000*	*0,000*	*0,000*
Chirurgische Nutzungszeit (min)	354,46	303,82	280,87	306,63
*Pro Tag pro Saal*	(100 %)	(85,71 %)	(79,24 %)	(86,51 %)
*p‑Wert*	*Referenz*	*0,000*	*0,000*	*0,000*
Wechselzeit (min)	32,86	34,69	40,13	44,37
*Pro Eingriff*	(100 %)	(105,57 %)	(122,12 %)	(135,03 %)
*p‑Wert*	*Referenz*	*0,003*	*0,000*	*0,000*
Operationszeit (min)	113,46	115,32	117,88	117,76
*Pro Tag pro Eingriff*	(100 %)	(101,64 %)	(103,90 %)	(103,79 %)
*p‑Wert*	*Referenz*	*0,220*	*0,003*	*0,002*
Startzeit (min)	21,08	37,39	56,67	41,72
*Pro Tag pro Saal*	(100 %)	(177,37 %)	(268,83 %)	(197,91 %)
*p‑Wert*	*Referenz*	*0,000*	*0,000*	*0,000*
Operationsschluss (min)	2,11	−8,41	−4,38	−16,37
*Pro Tag pro Saal*	(100 %)	(398,58 %)	(207,58 %)	(775,83 %)
*p‑Wert*	*Referenz*	*0,159*	*0,728*	*0,003*

In Tab. 3 ist zusätzlich die durchschnittliche Anzahl der Eingriffe pro Tag für die einzelnen Operationssäle über die Zeit aufgetragen. Die Säle wurden zwar auch teilweise multidisziplinär genutzt, der Rückgang der Operationszahlen während der Pandemie ist aber über alle Säle – wenn auch in unterschiedlicher Ausprägung – zu beobachten, was belegt, dass der Fallzahlrückgang alle analysierten Fächer betraf. Lediglich der überwiegend unfallchirurgische Operationssaal 6 hatte im Jahr 2022 das Operationsaufkommen des Jahres 2019 wieder erreicht.

**Tab. 3:** Operationsanzahl pro Tag und Saal in den Jahren 2019 bis 2022 – Mittelwerte

Jahr	2019	2020	2021	2022
Saal 1	2,55	2,34	2,47	2,32
	(100 %)	(91,76 %)	(96,86 %)	(90,98 %)
*p‑Wert*	*Referenz*	*0,071*	*0,410*	*0,015*
Saal 2	3,88	2,99	1,99	2,46
	(100 %)	(77,06 %)	(51,29 %)	(63,40 %)
*p‑Wert*	*Referenz*	*0,000*	*0,000*	*0,000*
Saal 3	3,39	3,13	2,71	2,88
	(100 %)	(92,33 %)	(79,94 %)	(84,96 %)
*p‑Wert*	*Referenz*	*0,047*	*0,000*	*0,000*
Saal 4	3,25	2,92	2,60	2,54
	(100 %)	(89,85 %)	(80,00 %)	(78,15 %)
*p‑Wert*	*Referenz*	*0,019*	*0,000*	*0,000*
Saal 5	3,86	3,71	3,62	3,37
	(100 %)	(96,11 %)	(93,78 %)	(87,31 %)
*p‑Wert*	*Referenz*	*0,132*	*0,029*	*0,000*
Saal 6	3,28	1,99	2,16	3,27
	(100 %)	(60,67 %)	(65,85 %)	(99,70 %)
*p‑Wert*	*Referenz*	*0,000*	*0,000*	*0,819*

### Korrelation zwischen COVID-19-Belegung des Krankenhauses und Operationssaalnutzung

#### Operationszahl

**Abb. 1:**
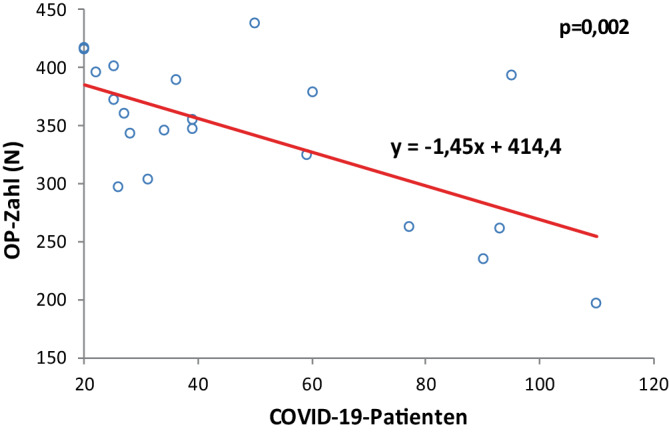
Korrelation zwischen Operationszahl und gleichzeitig im Krankenhaus behandelten COVID-19-Patienten

Mit steigender Zahl stationär behandelter COVID-19-Patienten nahm die Operationszahl signifikant ab (*p* = 0,002). Der Regressionskoeffizient beträgt −1,45, was bedeutet, dass pro zusätzlichem COVID-19-Patienten im Monat durchschnittlich 1,45 Operationen weniger durchgeführt wurden. Die Regressionsgleichung (y = −1,45x + 414,40) und die Verteilung der Datenpunkte belegen eine signifikante Beeinträchtigung der operativen Kapazitäten in Abhängigkeit von der Pandemiebelastung des Krankenhauses (Abb. 1).

#### Chirurgische Nutzungszeit

**Abb. 2:**
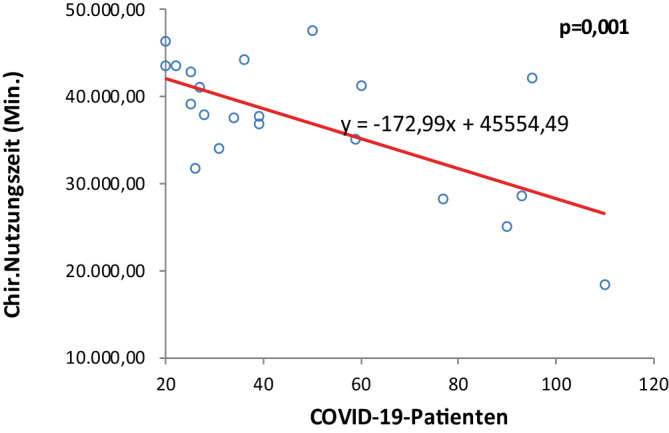
Korrelation zwischen chirurgischer Nutzungszeit und gleichzeitig im Krankenhaus behandelten COVID-19-Patienten

Den linearen Zusammenhang zwischen der Anzahl der COVID-19-Patienten im Krankenhaus und der gesamten chirurgischen Nutzungszeit pro Monat zeigt Abb. 2. Mit jedem zusätzlichen COVID-19-Patienten verringerte sich die chirurgische Nutzungszeit der Operationssäle im Durchschnitt um etwa 172,99 min (*p* < 0,001). Der Regressionskoeffizient ist statistisch signifikant (*p* < 0,01), was auf einen relevanten Einfluss der Pandemiebelastung auf die Operationskapazitäten schließen lässt.

#### Startzeit

**Abb. 3:**
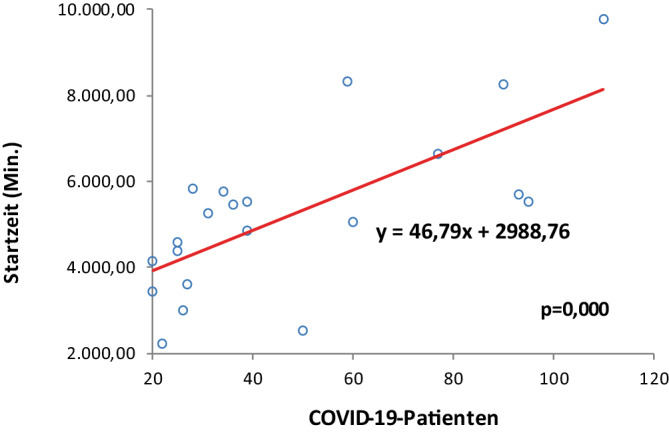
Korrelation zwischen Startzeit und gleichzeitig im Krankenhaus behandelten COVID-19-Patienten

Den Zusammenhang zwischen der Anzahl der COVID-19-Patienten im Krankenhaus und der verzögerten Operationsstartzeit veranschaulicht Abb. 3. Der positive Regressionskoeffizient von 46,79 deutet darauf hin, dass mit jedem zusätzlichen COVID-19-Patienten die verspätete Startzeit im Mittel um 46,79 min pro Monat ansteigt (*p* = 0,000). Die Ergebnisse legen nahe, dass sich organisatorische Engpässe, zusätzliche Hygienemaßnahmen und Personalverfügbarkeit erheblich auf den Operationsstart auswirkten.

## Diskussion

Die Auswirkungen der COVID-19-Pandemie auf das Management in der Allgemeinchirurgie ist vielfach beschrieben worden [2, 3, 9, 10, 13, 19], wobei speziell reduzierte Betten- und Operationssaalkapazitäten und Priorisierung der Eingriffe im Zentrum der Überlegungen standen, wie auch eine Umfrage bei Teilnehmern des Konvents der Ordinarien für Allgemein- und Viszeralchirurgie ergab [18].

Ähnliches gilt für die Herzchirurgie. Böning et al. [4] gaben an, dass sich mit Stand zum 30.04.2021 die herzchirurgische Intensivbettenkapazität in Deutschland um 25,6 % reduziert hatte, gleiches galt für die Anzahl der Operationssäle für herzchirurgische Eingriffe (−26,6 %). Eine internationale Umfrage in 61 herzchirurgischen Abteilungen [16] wies besonders neben der reduzierten Zahl operierter Patienten auf die Situation der Intensivstationen hin, von denen ein Drittel ausschließlich für die Behandlung von COVID-19-Patienten umgewandelt wurde.

Für die Gefäßchirurgie ist ein Bericht des britischen Vascular and Endovascular Research Network lehrreich, da er auf die veränderten Operationsindikationen hinwies [15]. Die Schwellenwerte des Aortendurchmessers für die Behandlung eines abdominellen Aortenaneurysmas (AAA) waren in 60 % der an der Umfrage teilnehmenden NHS-Zentren während der COVID-19-Pandemie erhöht, 16,7 % beschränkten die Operation auf einen AAA-Durchmesser von > 6 cm, 31,6 % auf > 7 cm und 23,3 % auf lediglich symptomatische oder rupturierte Aneurysmen. Jedes sechste AAA-Screening-Programm hatte seine Aktivitäten eingestellt. Eine ähnliche Beobachtung wurde in Frankreich bei Behandlung der Karotisstenose gemacht. In einer retrospektiven Kohortenstudie auf Basis der French-National-Hospital-Discharge-Datenbank identifizierten Crespy et al. [6] 12.546 Patienten, die zwischen Januar und September 2020 wegen operativen Eingriffen an der Karotis ins Krankenhaus eingeliefert wurden. Bei den Eingriffen handelte es sich um Karotisendarteriektomien (CEA) sowie transfemorales Karotisstenting (TFCAS). Im Vergleich zu den drei vorangegangenen Jahren zeigte sich während des Lockdowns ein signifikanter Rückgang bei Hospitalisierungen asymptomatischer Stenosen um 68,9 % (*p* < 0,001), bei den symptomatischen Stenosen hingen nur um 12,6 %. Nach Aufhebung des Lockdowns ergab sich bei CEA und TFCAS eine Erholung auf das Niveau der Vorjahre.

In der orthopädischen Chirurgie schließlich führte die frühe Pandemie im April bis Mai 2020 zu einem Rückgang der Operationszahlen um 36 % in der deutschen Wirbelsäulenchirurgie. Die elektiven Eingriffe erlebten eine Reduktion von 78 % auf 6 %. Die Operationsindikationen veränderten sich während der Pandemie zugunsten dringlicher (ca. 15 % vs. 52 %) und notfallmäßiger (ca. 7 % vs. 41 %) Eingriffe [20]. Eine Datenanalyse auf Basis des National-Joint-Registers (NJR) von Sabah et al. [17] ergab für das Jahr 2020 einen Rückgang bei Revisionsoperationen bei Kniegelenkersatz in Großbritannien um 43,6 % im Vergleich zum Vorjahr.

Das Operationsaufkommen während der COVID-19-Pandemie wurde allerdings mehrheitlich nur für relativ kurze Perioden beschrieben, nur eine Registererhebung bezog sich konkret auf einen längeren Erhebungszeitraum von 2018 bis 2022 [19]. Kaum bekannt ist, wie die COVID-19-Pandemie die Operationssaalnutzung und perioperativen Prozesszeiten über längere Zeit in einem Haus der Maximalversorgung veränderte. In Deutschland berichteten lediglich Datzmann et al. [7] für das Universitätsklinikum Ulm über verlängerte chirurgische Schnitt-Naht-Zeiten, Wechselzeiten und Anästhesiepräsenzzeiten während der COVID-19-Pandemie bis über die unmittelbaren COVID-Restriktionen hinaus im Jahr 2022. Zu einem ähnlichen Ergebnis kam nun die vorliegende Untersuchung: Es wurde im Vergleich zum Referenzjahr 2019 bis ins Jahr 2022 eine signifikant geringere Nutzung der Operationssäle über alle Fächer (Gefäßchirurgie, Viszeralchirurgie und Unfallchirurgie) beobachtet, mit verlängerten Wechselzeiten pro Eingriff, geringgradig, aber signifikant verlängerten Operationszeiten, einer späteren Operationsstartzeit und einem signifikant früheren Schluss des Regelbetriebs. Erhöhter perioperativer Hygieneaufwand und COVID-Schutzmaßnamen bieten eine Erklärung für verlängerte Eingriffszeiten und Wechselzeiten während der Pandemie. Gleichzeitig müssen die durch die Behandlung von COVID-Patienten eingeschränkten chirurgischen Betten- und Personalkapazitäten berücksichtigt werden, unter anderen auch aufgrund der Umverteilung von Mitarbeitern, wenn ärztliches und pflegerisches Personal (auch in der vorliegenden Untersuchung) in spezielle COVID-19-Bereiche delegiert wurde.

Erstmals wurde deshalb in dieser Untersuchung parallel zum Operationsaufkommen auch die Belegung des Krankenhauses mit COVID-19-Patienten erfasst. Die Analyse ergab eine signifikante inverse Korrelation zwischen Anzahl der im Krankenhaus behandelten COVID-Patienten und abnehmender Operationszahl und chirurgischer Nutzungszeit, verbunden mit verspäteter Startzeit, was die Ressourcenverschiebung eindeutig belegt. Hierfür spricht auch der Vergleich der Jahre 2020 und 2021. Der Rückgang des Fallvolumens war im Jahr 2020 mit 14,9 % geringer als im Jahr 2021 mit 23,1 %, bei gleichzeitig deutlich mehr stationär behandelten COVID-Patienten im Jahr 2021. Umgekehrt sahen Datzmann et al. [7] im Vergleich zu 2019 im Jahr 2020 mit 31 % einen stärkeren Rückgang des Fallvolumens als 2021 mit 20 %.

Über die Operationssaaleffizienz eines orthopädischen Zentrums (Mailand) unter COVID-Bedingungen berichteten Andreata et al. [1], wobei sie die Leistungen im April 2020 mit denen im April 2019 (vor COVID-19) miteinander verglichen. Sie nannten einen Rückgang der Eingriffszahlen von 537 im Jahr 2019 auf 230 in 2020 (57 %) und eine Verspätung des ersten Eingriffs am Tag von 2 h und 36 min im Vergleich zu 19 min vor Corona. Die Wechselzeiten erhöhten sich von 21 min im Jahr 2019 auf 53 min im Jahr 2020. Als Gründe für die Verzögerungen gaben sie das Warten auf das Ergebnis des SARS-CoV-2-Mundabstrichs, Abschlussuntersuchungen und Sicherheitsverfahren, Wechsel des Operationsteams, Neuorganisation der Operationsteams und Planungsschwierigkeiten an. Darüber hinaus war das Anlegen der persönlichen Schutzausrüstung extrem zeitaufwendig. Inwieweit allerdings die Analyse von nur 1 Monat repräsentativ ist, muss offenbleiben. In der vorliegenden Untersuchung wurde deshalb über 4 Jahre die Operationssaaleffizienz jeweils über 7 Monate (Januar bis Juli) erfasst.

Eine weitere Untersuchung einer orthopädischen Schwerpunktklinik (Nashville) [12] zur Operationssaaleffizienz unter COVID-19-Bedingungen unterschied zwischen drei Perioden: Prä-COVID (90 Tage vor März 12, 2020, *n* = 617), COVID-Spitze (90 Tage nach März 12, 2020, *n* = 442) und Zeit nach Aufhebung der Restriktionen bis Oktober 31, 2021 (*n* = 3854). In dieser Untersuchung gab es zwischen den Zeiträumen nur geringe, wenn auch signifikante Unterschiede im Operationsstart (Verzögerung im Mittel lediglich 7–8 min). In der Zahl der Fälle, die eine Verzögerung um mehr als 15 min aufwiesen, unterschieden sich die drei Zeiträume nicht (16 %, 18 %, 16 %). Auch waren die Wechselzeiten klinisch nicht relevant, wenn auch statistisch signifikant unterschiedlich (im Mittel 62, 66 und 64 min). Die Autoren folgerten, dass die Operationssaaleffizienz in ihrer Institution unter COVID-Bedingungen aufrechterhalten werden konnte, was sie auf die Einführung spezifischer Ablaufpfade zurückführten. Nicht analysiert wurden allerdings die Kapazitäten (Fallzahlen) des Hauses unter COVID-19-Bedingungen. Eine Untersuchung aus Großbritannien demonstrierte unter COVID-19-Bedingungen eine signifikante Reduktion der Operationssaaleffizienz für traumatologische Behandlungsfälle im Vergleich von 2019 zu 2020 [14]. Die Operationszeit stieg 2020 signifikant (*p* < 0,025) von im Mittel 54,40 (47,44–61,36) auf 61,75 (48,73–74,76) min an, die Wechselzeit sogar von 31,77 (23,02–40,52) auf 57,42 (42,00–72,83) min (*p* = 0,000). Interessanterweise war aber die Anästhesiezeit (Zeit von Beginn der Anästhesieeinleitung bis Eintritt des Patienten in den Operationsraum) unter COVID-Bedingungen verkürzt, was die Autoren mit der Reduzierung der Kombinationsanästhesien (Allgemeinanästhesie + Regionalanästhesie) erklärten.

Die Ergebnisse der vorliegenden Studie zeigen, dass externe Krisensituationen wie die COVID-19-Pandemie nicht nur zu einem Rückgang des Operationsvolumens führen, sondern auch erhebliche Auswirkungen auf zentrale Prozessparameter haben. Verzögerte Operationsstartzeiten, verlängerte Wechselzeiten und reduzierte chirurgische Nutzungszeiten verdeutlichen, wie verletzlich bestehende Strukturen unter außergewöhnlicher Belastung sind. Diese Erkenntnisse haben sowohl auf Klinikebene als auch gesundheitspolitisch relevante Konsequenzen.

Auf Klinikebene wird deutlich, dass Operationsplanungssysteme krisenresilienter gestaltet werden müssen. Flexible Kapazitätsmodelle, die kurzfristige Umverteilungen von Personal und Ressourcen erlauben, sind essenziell. Standardisierte Prozesspfade sollten überarbeitet und für Szenarien mit hoher Belastung angepasst werden, um Effizienz und Sicherheit zu gewährleisten. Darüber hinaus spielt die Koordination zwischen chirurgischen, anästhesiologischen und pflegerischen Teams eine Schlüsselrolle, um Verzögerungen zu minimieren und Engpässe frühzeitig zu erkennen. Digitale Operationsmanagementsysteme, die Echtzeitdaten aus Intensiv- und Normalstationen integrieren, könnten hier einen entscheidenden Beitrag leisten.

Gesundheitspolitisch lässt sich aus den Daten ableiten, dass eine stärkere regionale und sektorübergreifende Planung notwendig ist. Die inverse Korrelation zwischen COVID-19-Belegung und Operationsleistungsparametern zeigt, dass die Auslastung einzelner Krankenhausbereiche – insbesondere der Intensivstationen – den Operationsbetrieb direkt beeinflusst. Für zukünftige Pandemien oder ähnliche Krisensituationen könnten daher Lastverteilungsmechanismen etabliert werden, die eine gezielte Entlastung einzelner Kliniken ermöglichen. Ein denkbares Konzept wäre die Definition von COVID-Schwerpunktkliniken, um elektive Operationskapazitäten in anderen Häusern aufrechtzuerhalten und eine gleichmäßigere Verteilung der Belastung zu gewährleisten.

Die in dieser Arbeit dargestellten Ergebnisse zum Rückgang der Operationszahlen und zur Veränderung zentraler Prozessparameter stehen in Übereinstimmung mit Daten des DIVI-Intensivregisters und des Robert Koch-Instituts (RKI). Das DIVI-Register erfasst seit April 2020 verpflichtend die intensivmedizinische Belegung und Bettenkapazitäten in über 1300 deutschen Akutkrankenhäusern. Die Daten dokumentierten die pandemiebedingten Belastungsspitzen – insbesondere während der Winterwellen 2020/2021 und 2021/2022 – und zeigten die parallele Abnahme freier ITS-Betten, häufig unter die kritische 10 %-Grenze [8].

Zu den Limitationen dieser Untersuchung gehört, dass Diagnosen und Prozeduren nicht erfasst werden konnten, sodass über ein verändertes Operationsspektrum unter COVID-Bedingungen keine konkreten Aussagen gemacht werden können. Auch wurden die Jahre 2019 bis 2022 nicht vollständig erfasst, sondern – um den Dokumentationsaufwand zu begrenzen – jeweils die ersten 7 Monate der Jahre 2019 bis 2022. Die Konzentration auf die ersten 7 Monate erlaubt eine gleichlange Beobachtungsperiode pro Jahr, die sowohl vorpandemische (2019) als auch pandemiebedingte Belastungsphasen (2020–2022) einschließt. Zwar bleiben dadurch die Effekte der ausgeprägten Herbst‑/Winterwellen teilweise unberücksichtigt, dennoch lassen sich durch die gewählte Zeitspanne robuste und konsistente Trends in den Operationskennzahlen ableiten. Alle in dieser Arbeit zitierten Studien untersuchten einen deutlich kürzeren Zeitraum.

In dieser Analyse wurde zwischen Notfall- und Planeingriffen nicht unterschieden, da dies den Dokumentationsaufwand gesprengt hätte. Ziel der Arbeit war es, die Auswirkungen der COVID-19-Pandemie auf die Auslastung und Prozessparameter der Operationssäle zu untersuchen – also den Fokus gezielt auf die strukturellen und organisatorischen Veränderungen im Operationsbetrieb zu legen. Die signifikante Reduktion der Operationszahlen, insbesondere in den Jahren 2020 und 2021, ist aber vor allem auf die Verschiebung elektiver Eingriffe, die Freihaltung von Kapazitäten für Notfälle und COVID-19-Patienten sowie auf die Umwidmung einzelner Operationssäle (z. B. Saal 6 – wurde Teil der Intensivstation in den Jahren 2020 und 2021) zurückzuführen. Obwohl die Gesamtoperationszeit zurückging, blieben die durchschnittlichen Operationsdauern pro Eingriff weitgehend stabil. Dies spricht dafür, dass das chirurgische Spektrum erhalten blieb. Es wurden keine Hinweise auf eine systematische Verkürzung der Eingriffe gefunden; vielmehr waren es die fallzahlbedingten Reduktionen, die den Rückgang des Gesamtvolumens verursachten. Der deutliche Rückgang der chirurgischen Nutzungszeit lässt sich ebenfalls mit reduziertem Operationsvolumen und Personalausfällen erklären. Durch die Verlagerung des Operationsfokus auf dringliche Eingriffe wurden verfügbare Kapazitäten zwar genutzt, jedoch insgesamt auf einem niedrigeren Niveau. Dies ist Ausdruck sowohl der pandemiebedingten Einschränkungen als auch einer strategischen Reduktion planbarer Operationen.

Zusammenfassend gibt diese Untersuchung, in der bei 10.193 jeweils konsekutiven Eingriffen die Belegung des Operationssaals, Benutzungszeiten und Wechselzeiten erfasst werden konnten, einen kaum berichteten Einblick in den Einfluss der COVID-19-Pandemie auf das Operationsmanagement einer Klinik. Einmalig ist darüber hinaus, dass hier der Einfluss der COVID-19-Behandlungsfälle im gesamten Klinikum mit dem Operationsvolumen und Operationsaufwand korreliert werden konnte.

## Fazit für die Praxis

- Die COVID-19-Pandemie führte nicht nur – verglichen mit dem Jahr 2019 – mit Beginn des ersten strengen Lockdowns im März 2020, sondern bis in das Jahr 2022 zu einer signifikanten Reduktion des Operationsaufkommens, verringerter Operationssaalnutzung und erhöhten Wechselzeiten in einem Haus der Maximalversorgung bei gefäßchirurgischen, viszeralchirurgischen und unfallchirurgischen Eingriffen.
- Eine signifikante Korrelation zwischen Belegung des Krankenhauses mit COVID-19-Patienten und reduzierter Operationseffizienz konnte gezeigt werden.

## Data Availability

Die Auswertung beruht auf dem Klinikdokumentationssystem, alle verwendeten Daten sind anonym abrufbar. Die Daten können auf Anfrage bereitgestellt werden. Alle relevanten Daten sind im Artikel enthalten.
